# Novel Strategies Using Total Gastrodin and Gastrodigenin, or Total Gastrodigenin for Quality Control of *Gastrodia elata*

**DOI:** 10.3390/molecules23020270

**Published:** 2018-01-29

**Authors:** Chunlan Tang, Bingchu Wu, Jinyi Wu, Zheng Zhang, Bocheng Yu

**Affiliations:** Department of Preventative Medicine, Medical School of Ningbo University, Ningbo 315211, China; bingchu97@163.com (B.W.); wujiny@foxmail.com (J.W.); ZZ1171987191@163.com (Z.Z.); a13757451117@163.com (B.Y.)

**Keywords:** total gastrodin, total gastrodigenin, base-enzymatic hydrolysis, HPLC-FLD

## Abstract

*Gastrodia elata* Blume (*G. elata*), a traditional Chinese medicine, is widely used for treatment of various neuro dysfunctions. However, its quality control is still limited to the determination of gastrodin. In the present study, two novel strategies based on quantitative evaluation of total gastrodin and gastrodigenin with base hydrolysis and total gastrodigenin with base-enzymatic hydrolysis followed by HPLC-FLD were put forward and successfully applied to evaluate the quality of 47 batches of *G. elata* from eight localities. Meanwhile, a systematic comparison of the novel strategy with the multiple markers and the Pharmacopeia method was performed. The results showed that the parishins category could be completely hydrolyzed to gastrodin by sodium hydroxide solution, and gastrodin could further utterly hydrolyze to gastrodigenin with β-d-glucosidase buffer solution. The contents of total gastrodin and gastrodigenin ranged from 1.311% to 2.034%, and total gastrodigenin from 0.748% to 1.120% at the eight localities. From the comparison, we can conclude that the two novel strategies can comprehensively reveal the characteristics of overall active ingredients in *G. elata* for quality control. The present study provides a feasible and credible strategy for the quality control of *G. elata*, suggesting a revision of the latest Chinese Pharmacopoeia or European Pharmacopoeia methods for the modernization of *G. elata* use.

## 1. Introduction

*Gastrodia elata* Blume (*G. elata*), the tuber of the orchid, is a traditional Chinese medicine (TCM) extensively used against convulsions, vertigo, paralysis, epilepsy, tetanus, asthma and immune dysfunctions [1,2,3,4,5,6]. Gastrodin (GAS) is widely considered as the main and bioactive ingredient in *G. elata* for its sedative, anticonvulsive and neuroprotective effects [7,8,9,10]. Therefore, a large number of studies on the pharmacokinetics, and pharmacological studies of GAS have been carried out [11,12,13]. In general, TCMs contain a large number of ingredients, many of which exert favorable pharmacological activity [14,15]. Therefore, choosing only one or two ingredients as markers for quality control of TCM is not accord with the holistic characteristics of TCMs.

With the development of phytochemistry, a lot of active ingredients, including gastrodigenin (*p*-hydroxybenzyl alcohol, HBA), the parishins, nucleosides, etc. were identified in *G. elata* [16,17], and these ingredients revealed preferable efficacy [18,19], particularly in terms of neuroprotective efficacy [20,21,22,23]. Therefore, the use of GAS as the single marker is not enough for the quality control of *G. elata*. In the Chinese Pharmacopoeia (2015 version), HBA was added as another marker, that is, the GAS and HBA both control the quality of *G. elata*. In addition, Li et al. reported the use of 5-hydroxy-methyl-2-furaldehyde, parishin B (PB) and parishin C (PC) as markers of *G. elata* by fingerprint-efficacy relationship modelling [24]. Ma et al. employed GAS, HBA, parishin E (PE), PB, PC and parishin (PA) as markers to evaluate the quality of *G. elata*. However, as reported, *G. elata* contains almost eighty ingredients [25]. Among them, some exert good and various pharmacological activity, so only choosing GAS, HBA or PC as markers is not enough to cover most ingredients and explain the overall characteristic of *G. elata*, such as its pharmacokinetic or pharmacodynamic properties [26]. Therefore, some researchers develop the use of fingerprint methods or multi-component patterns for the quality control and origin discrimination of *G. elata* by similarity index [27,28,29]. Nevertheless, fingerprint evaluation of TCM quality of still has many challenges, including how to definite the common peaks, how to solve the problems of background drift, overlapping peaks, noisy peaks, etc.

The present study aimed to investigate two novel strategies for quality control of *G. elata*, which could comprehensively reveal the characteristics of overall active ingredients in *G. elata*, and could be conveniently and effectively adapted in various laboratory and clinical applications. Therefore, two novel strategies, based on quantitative evaluation of total GAS and HBA with base hydrolysis (TGH-B), and total HBA with base-enzymatic hydrolysis (TH-BE) followed by HPLC-FLD techniques, were carried out and applied to evaluate 47 batches of *G. elata* from eight localities. In addition, the comparison of the proposed strategies with multiple markers and the Pharmacopeia method for quality control of *G. elata* was also investigated [30,31]. The success of the novel strategy suggest a revision of the Pharmacopoeia method and lays the foundation for the modernization of *G. elata* use.

## 2. Results

### 2.1. Development of the Method

**Figure 1:**
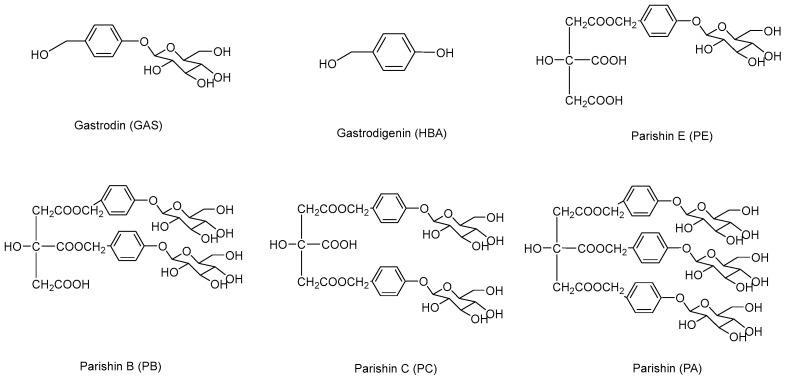
The structures of gastrodin (GAS), gastrodigenin (HBA), parishin E (PE), parishin B (PB), parishin C (PC) and parishin (PA).

**Figure 2:**
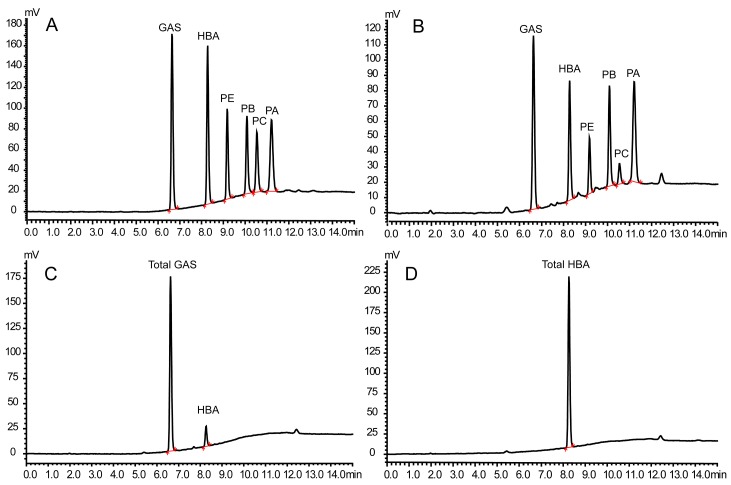
Representative chromatograms: (**A**) standard solution including GAS, HBA, PE, PB, PC and PA; (**B**) extract of *G. elata*; (**C**) extract of *G. elata* after base hydrolysis; (**D**) extract of *G. elata* after base-enzymatic hydrolysis. The injected volume of extract of *G. elata* was 10 μL, and the hydrolyzed samples was 2 μL.

Our previous studies have reported the fluorescence properties of the main phenols in *G. elata* [32]. Considering the different instrumentation, the excitation and emission wavelength were further optimized by scanning the fluorescence spectrum of each compound. The results showed that the maximum excitation and emission wavelengths were at 275 nm and 295 nm, respectively. In addition, the flow rate, column temperature and gradient elution condition were optimized to achieve well separated and sharp peaks. After optimization of the chromatographic conditions, six compounds, including GAS, HBA, PE, PB, PC and PA (Figure 1), were effectively separated in 15 min when the column was eluted in a gradient of methanol and water containing 0.5% formic acid. The retention times of GAS, HBA, PE, PB, PC and PA were 6.64, 8.27, 9.17, 10.07, 10.53, 11.20 min, respectively (Figure 2A).

The regression curve presented high linearity, with a coefficient (*r*^2^) larger than 0.99 within the concentration range from 0.05 to 10 μg/mL, demonstrating its suitability for quantitative analysis of GAS, HBA, PE, PB, PC, PA. The limit of quantitation (LOQ) for six compounds was 0.05 μg/mL. The RSD and RE at three concentration levels (five replicates) of GAS, HBA, PE, PB, PC, PA were all within ±15%, indicating the good precision and trueness.

The above results are summarized in Table 1. In addition, the extraction recoveries and matrix effects of GAS, HBA, PE, PB, PC and PA were 95.28%, 92.20%, 110.27%, 114.98%, 99.52%, 101.32% and 94.48%, 97.63%, 101.93%, 100.79%, 106.79%, 103.84%, respectively (Table 2). These results indicated that the present method was reliable and reproducible for the quantitative analysis of GAS, HBA, PE, PB, PC and PA.

### 2.2. Quality Evaluation by Pharmacopoeia Indices

GAS and HBA are used as quality guideline of *G. elata* in the Chinese Pharmacopoeia (2015 version), where the added content of GAS and HBA was set as not less than 0.25%. GAS is used as quality criterion in the European Pharmacopoeia, where the content of GAS is set as not less than 0.20%. Therefore, this study firstly determined the content of GAS and HBA of 47 batches of *G. elata* from eight localities using the developed method. The results showed that the added content of GAS and HBA at the eight localities ranged from 0.181% to 0.942%, while GAS was 0.106% to 0.918%, which almost meets the criteria of both pharmacopoeias, except for seven samples which were marked in red in Supplementary Material Table S1. The above results indicated that the quality of *G. elata* from various drugstores are almost compliant with the guidelines. In addition, the quality of *G. elata* from eight localities was sorted according to the average of content sum of GAS and HBA or the single GAS, the order was as follows: Anhui > Xizang> Zhejiang > Sichuan > Yunnan > Guizhou > Jilin > Shanxi (Table 3).

### 2.3. Quality Evaluation by Multi-Markers

**Figure 3:**
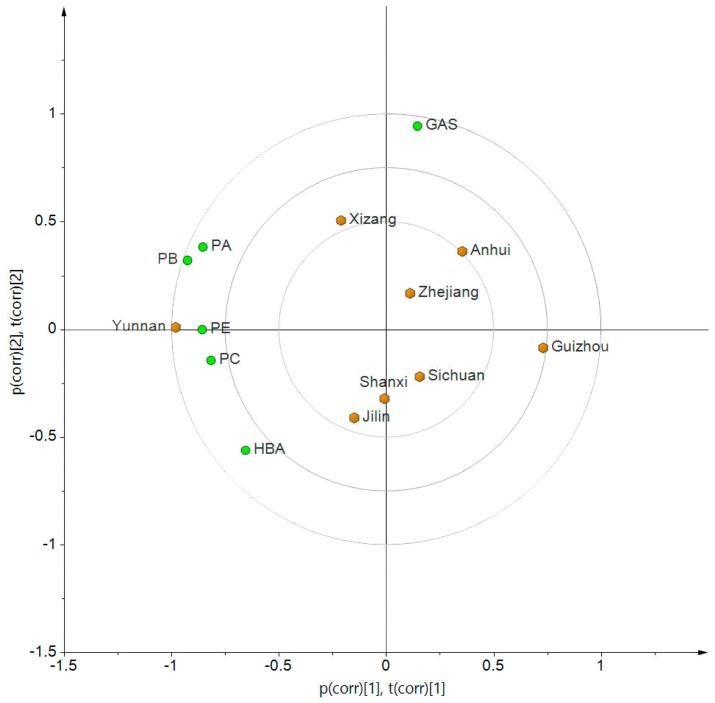
Biplot of the average content of GAS, HBA, PE, PB, PC and PA with eight localities of *G. elata*.

The fluorescence fingerprint chromatograms of *G. elata* extract were produced and are depicted in Figure 2B. A total of six peaks which were assigned as GAS, HBA, PE, PB, PC and PA, respectively, were unequivocally identified by comparing their retention times with those of reference substances. In addition, the results showed that the six active compounds were all detected at all batches of *G. elata* from eight localities. The quantitative analysis results showed that the average content of PE, PB, PC and PA was highest at Yunnan region, GAS at Anhui region, and HBA at Sichuan region. In addition, the correlation analysis of locality and content of active compounds was carried out using the SIMCA 14.1 software (Umetrics, Umea, Sweden). As seen from Figure 3, it can be obtained that the four parishins and Yunnan region were almost at the same position. The feature distinguishing the Yunnan region from the other seven regions was the parishins. Shanxi, Sichuan and Jinlin can be classified into the same cluster and were close to the HBA, indicating that they all possess relative higher contents of HBA. Anhui, Zhejiang and Xizang could be placed into the same group, which possesses a relatively high content of GAS. In addition, Guizhou region is the furthest from the four parishins, showing the lowest content of these compounds. The total content of the six active compounds in *G. elata* from the eight localities ranged from 2.274% to 3.700% (Table 2). According to the total content, the quality of *G. elata* from the eight localities could be ranked as follows: Yunnan > Xizang > Zhejiang > Jilin > Anhui > Shanxi > Sichuan > Guizhou.

### 2.4. TGH-B Strategy by Total GAS and HBA

**Figure 4:**
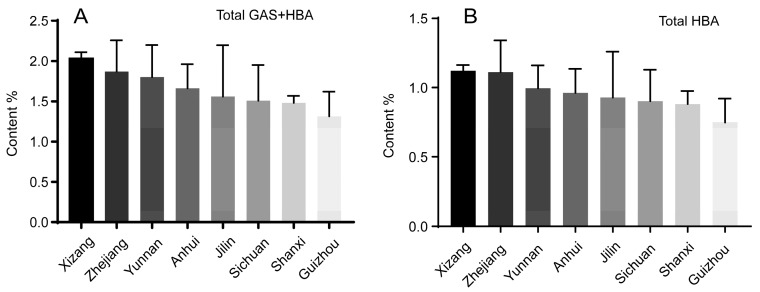
The content of components of *G. elata* after hydrolysis: (**A**) the sum of total GAS and HBA in *G. elata* after base hydrolysis; (**B**) total HBA in *G. elata* after base-enzymatic hydrolysis. Data, expressed as percentage of crude drug, were the mean ± SD of different batches, and each batch at three experiments.

Our previous study showed that PA could hydrolyze to GAS [33], and Li et al. reported that there are over twenty parishins in *G. elata* [34]. In addition, considering the difficulty of preparation of the parishins and the instability of the parishins, this study adopted a hydrolysis experiment, whereby the parishins were hydrolyzed to GAS by an alkaline solution, and then the total GAS and HBA was determined after base hydrolysis for quality evaluation. The hydrolytic condition including temperature (60–80 °C), time (0.5–2 h) and alkali concentration (0.5–3 mol/L) were optimized. The results showed that no remarkable variation was observed under the above-mentioned conditions. Therefore, the temperature of 80 °C, time of 0.5 h and alkali concentration of 0.5 mol/L were chosen as hydrolytic conditions. Under these conditions, the samples were hydrolyzed and further analyzed. Comparing the chromatograms of hydrolysis before and after (Figure 2A,C), all peaks of parishins have disappeared and the peak of GAS was remarkably increased, indicating that the parishins were all completely hydrolyzed under the present conditions. Then the content of total GAS and HBA were determined, which were ranged from 1.311% to 2.034% for the eight localities. According to the content sum of total GAS and HBA, the quality of *G. elata* from the eight localities followed the order Xizang > Zhejiang > Yunnan > Anhui > Jilin > Sichuan > Shanxi > Guizhou (Figure 4A). 

### 2.5. TG-BE Strategy by Total HBA

Many studies have reported that GAS could metabolize to HBA, and HBA could cross the blood-brain barrier and exert its neuroprotective effects [35,36,37]. Therefore, this study hydrolyzed the parishins to produce GAS by alkaline solution, and further hydrolyzed GAS to HBA by enzyme solution, employing the total HBA level as a quality index. Firstly, the condition of enzymatic hydrolysis was investigated by L9(3^4^) orthogonal experiment. The orthogonal experiment result showed that GAS could be totally hydrolyzed to HBA at an enzyme concentration of 200 U, rotation speed of 1000 rpm, and a time of 3 h (see Supplementary Material Table S2). Under these conditions, all batches of extracts of *G. elata* were hydrolyzed and then further analyzed using HPLC-FLD. From the fluorescence chromatogram (Figure 2D), it can be obtained that a single HBA peak was detected and the peak height was observably increased, suggesting that the parishins and GAS were all utterly decomposed to HBA. The content of total HBA ranged between 0.748% and 1.120% for the eight localities. According to the content of total HBA, we can rank the quality of *G. elata* from the eight localities in the following order: Xizang > Zhejiang > Yunnan > Anhui > Jilin > Sichuan > Shanxi > Guizhou (Figure 4B).

## 3. Discussion

Many previous studies mainly used GAS as the marker of *G. elata*, and a large number of studies on the pharmacokinetics, and pharmacological studies of GAS were carried out [38,39,40]. With the development of phytochemistry, a lot of active compounds, including HBA, the parishins, the nucleosides, etc. were identified, so the use of GAS as the single marker was not enough for the quality control of *G. elata*. In the latest version of Chinese Pharmacopoeia in 2015, HBA was added as another marker, that is, the GAS and HBA both control the quality of *G. elata*, but as we all know, each TCM usually contains hundreds of ingredients. Among them, some exert good and varied pharmacological activity, so only choosing one or a few of the ingredients as markers is not enough to explain the overall characteristics of a TCM. Therefore, two novel TGH-B and TH-BE strategies using the sum of total GAS and HBA, and total HBA were proposed and successfully applied for quality evaluation of *G. elata* in 47 batches from eight localities. A comparison between the TGH-B and TH-BE strategies with the Pharmacopoeia method and multiple makers method was carried out. The results are shown in Table 4. We can observe that the results of determination of total GAS and HBA or total HBA were basically identical, which may be on account of two reasons. One is that GAS was thoroughly hydrolyzed to HBA by β-d-glucosidase enzymes. Another is that few isomers of GAS, which could be hydrolyzed to HBA by β-d-glucosidase, exist in *G. elata*, or the isomers of GAS could not convert into HBA by enzymatic hydrolysis. Therefore, the order of the content of total GAS and HBA, and total HBA was basically consistent for the eight localities. However, comparing the multiple markers method with the TGH-B and TH-BE strategies, a few turned out different. It has been reported that over 20 kinds of parishins such as parishin K, parishin N, etc. are found in *G. elata* [34], not only PE, PB, PC and PA. According to the structure of the parishins, these compounds may all hydrolyze to GAS, so if only four parishins are used, the content of other parishins is not considered, which resulted in the differences observed. Homoplastically, if only GAS and HBA, or only GAS are determined, many active ingredients are not considered, which could not represent the overall properties and result in a quite different order from the other three methods. The novel strategies by determination of total GAS and HBA, or total HBA after base or base-enzymatic hydrolysis, covered most active ingredients, which all possessed the active *p*-hydroxybenzyl alcohol structure and could be hydrolyzed by alkali or enzymatic solutions. Therefore, the two strategies can comprehensively reflect the content of total active ingredients in *G. elata*, which could provide better scientific strategies for the quality control of *G. elata*, and suggest that a revision of the latest Chinese or European pharmacopoeias for the modernization of *G. elata* use should be considered.

## 4. Materials and Methods

### 4.1. Chemicals and Reagents

Eight localities in China were selected and 3–10 batches of *G. elata* were purchased at each locality from various pharmacies (see Supplementary Material Table S3). Voucher specimens were deposited in the Key Laboratory of Pathological and Physiological Technology, Ningbo, Zhejiang. Parishin (PA > 98%), parishin B (PB > 95%), parishin C (PC > 95%), and gastrodigenin (HBA > 98%) was isolated and purified from the dried roots of *G. elata* and identified in our lab [15,25]. Gastrodin (GAS > 98%) and parishin E (PE > 95%) were purchased from Chengdu Biopurify Phytochemicals Co., Ltd. (Chengdu, China). β-d-Glucosidase was purchased from Wuhan Dahua Pharmaceutical Co., Ltd. (Wuhan, China). Methanol of chromatographic grade were obtained by Tedia (Fairfield, OH, USA). Formic acid of chromatographic grade, sodium hydroxide, sodium acetate and acetic acid of analytical grade were purchased from Aladdin (Shanghai, Chuina). Calibration solution of pH 4.1 was purchased from Shanghai Solarbio Life Science Co., Ltd. (Shanghai, China). Hydrochloric acid of analytical grade was purchased from Zhejiang Zhongxing Chemical Reagent Co., Ltd. (Zhejiang, China). Ultrapure water was obtained by a water purification system (Milli integral 5, Millipore, Billerica, MA, USA).

### 4.2. Instrumentation

The chromatographic analysis was carried out on an HPLC-FLD system (Shimadzu, Kyoto, Japan), equipped with a pump, autosampler, degasser, column oven and fluorescence detector. Separation was performed on a Shimadzu reverse phase InertSustain C18 HPLC column (4.6 × 150 mm, 5 μm) with column oven 30 °C. The elution gradient was composed of 0.5% formic acid aqueous solution (C) and methanol (A) at a flow rate of 1 mL/min. Optimized separation of targets was obtained according to a linear gradient that increased from 5% to 40% A in 5 min and held for additional 10 min. The injection volume was set at 10 μL for extracted sample and 2 μL for hydrolytic sample. The excitation and emission wavelengths of the fluorescence analysis were set at 275 nm and 295 nm, respectively.

### 4.3. Preparation of Standard Solution and Extract of G. elata

PA, PB, PC, PE, GAS and HBA powders were separately dissolved in 50% methanol-water at a final concentration of 1 mg/mL. Calibration standard solutions were prepared by serial dilution of primary stock solution with 50% methanol-water to obtain concentrations of 0.05, 0.1, 0.5, 1, 5 and 10 μg/mL, respectively. Quality control (QC) samples were prepared at 0.2 μg/mL, 2 μg/mL and 6 μg/mL of low, medium and high concentration levels, which were used in developed analytical methods. All solutions were kept at 4 °C in fridge and were brought to room temperature prior to use. The dried tubers of *G. elata* were finely powered, and 0.5 g of the powder was precisely weighed and extracted with 50% methanol-water (1:20, *w*/*v*) for 1 h by ultrasound. After extraction, the mixture was centrifuged at 3000 rpm for 10 min. The supernatant was collected and filtered by 0.22 μm filter membrane. 5 μL of filtrate was dilute with 50% methanol-water to 1 mL, A 10 μL aliquot was injected for HPLC-FLD analysis.

### 4.4. Hydrolysis

#### 4.4.1. Base Hydrolysis

*G. elata* extract (50 μL) was added with 1 mL of 0.5 mol/L sodium hydroxide solution. Sample solutions were maintained at 80 °C for 0.5 h in a water bath. After hydrolysis, the sample was neutralized with 0.5 mol/L hydrochloric acid solution, and diluted with 50% methanol-water to 2 mL. 200 μL of sample was taken and added 800 μL of 50% methanol-water. A 2 μL aliquot was injected for HPLC-FLD analysis.

#### 4.4.2. Base-Enzymatic Hydrolysis

Sodium acetate (5.4 g) was dissolved in water (50 mL), and the pH adjusted to 4.60 with acetic acid in a 100 mL volumetric flask to prepare sodium acetate buffer solution. β-d-Glucosidase was dissolved in sodium acetate buffer solution. Fifty μL of *G. elata* extract was added with 50 μL of 0.5 mol/L sodium hydroxide solution. Sample solutions were maintained at 80 °C for 0.5 h in a water bath. After hydrolysis, the sample was neutralized with 0.5 mol/L hydrochloric acid solution, then added 500 μL of 4 mg/mL of β-d-glucosidase solution, the final volume was 1 mL. Sample solutions were incubated for 3 h in a vortex mixer at 1000 rpm. After incubation, the sample was heated at 85 °C for 4 min in a water bath, and 3 mL of ice-cold methanol was added to precipitate the proteins and vortex-mixed for 5 min when cooled. Then the samples were centrifuged at 12,000 rpm for 10 min at 4 °C. The supernatant was transferred and filtered by 0.22 μm filter membrane. 400 μL of filtrate was taken and added with 600 μL of 50% methanol-water solution for analysis. A 2 μL aliquot was injected for HPLC-FLD analysis.

### 4.5. Analytical Method Validation

#### 4.5.1. Linearity and LOQ

Calibration curves was plotted from standard samples in the concentration range of 0.05–10 μg/mL by plotting the peak area (*y*) versus concentration (*x*) of each analyte. The LOQ were defined as the concentration of lowest concentration standard in the calibration curve that was analyzed with a precision (RSD %) not exceeding 20% and with a trueness between 80% and 120%.

#### 4.5.2. Precision and Trueness

The precision and trueness were determined by analyzing QC samples for three concentration levels. Each level was analyzed five replicates within 1 day for intra-day precision and trueness, while once a day during five consecutive days for inter-day precision and trueness. Precision was expressed as relative standard deviation (RSD), and trueness was calculated by relative error (RE) at each concentration level. For trueness and precision, the criterion for the acceptability of data should not deviate by ±15% from the nominal concentration.

#### 4.5.3. Recovery of Extraction and Matrix Effect

The GAS, HBA, PE, PB, PC and PA were added into the power sample and the extract of *G. elata* for determination of recovery of extraction and matrix effect, respectively. The spiked concentration of GAS, HBA, PE, PB, PC and PA was 0.5 μg/mL, three duplicates of the mixed samples were further analyzed, respectively. The extraction recovery was determined by comparing the calculating concentration following extraction and HPLC assays with the spiked concentration. The matrix effect was calculated by comparing the different value of peak area between determined and origin value of extract with that of 50% methanol-water solution. 

## 5. Conclusions

At the present study, two novel strategies for quality control of *G. elata* were proposed and successfully applied to evaluate the quality of 47 batches of *G. elata* from eight localities. A comparison of the proposed strategies with the Pharmacopoeia and multiple markers methods was also investigated. The results showed that the novel TGH-B and TH-BE strategies, which determined the total GAS and HBA, and total HBA, could comprehensively reflect the content of total active ingredients in *G. elata*, providing an effective strategy for the quality control of *G. elata*, and suggesting the revision of the latest Chinese or European pharmacopoeias for the modernization of *G. elata* use.

## Figures and Tables

**Table 1:** Intra- and inter-day precision and trueness, linearity and limit of quantitation (LOQ) of gastrodin (GAS), gastrodigenin (HBA), parishin E (PE), parishin B (PB), parishin C (PC) and parishin (PA).

Compounds	Precision and Trueness (*n* = 5)							Linearity	LOQ
	Concentration (μg/mL)	Intra-Day			Inter-Day				
		Mean ± SD	RSD (%)	RE (%)	Mean ± SD	RSD (%)	RE (%)		
GAS	0.2	0.19 ± 0	0.94	−5.05	0.18 ± 0	3.37	−6.67	*y* = 9.12 × 10^5^*x* + 6.69 × 10^4^	0.5
	1.2	1.18 ± 0	0.20	−1.70	1.16 ± 0.02	1.85	−3.50		
	6	5.97 ± 0.02	0.36	−0.46	5.86 ± 0.11	1.89	−2.37		
HBA	0.2	0.22 ± 0	0.20	9.58	0.21 ± 0	2.27	6.55	*y* = 2.07 × 10^6^*x* − 7.44 × 10^4^	0.5
	1.2	1.19 ± 0	0.29	−0.48	1.16 ± 0.03	2.41	−3.08		
	6	5.97 ± 0.01	0.18	−0.47	5.75 ± 0.14	2.42	−4.10		
PE	0.2	0.17 ± 0	3.55	−14.97	0.17 ± 0	3.00	−14.77	*y* = 1.65 × 10^5^*x* − 6.01 × 10^3^	0.5
	1.2	1.10 ± 0.02	1.41	−8.64	1.06 ± 0.03	2.95	−12.02		
	6	5.60 ± 0.03	0.46	−6.73	5.45 ± 0.11	2.10	−9.11		
PB	0.2	0.17 ± 0	5.10	−14.16	0.17 ± 0	2.33	−13.19	*y* = 2.16 × 10^5^*x* + 5.31 × 10^3^	0.5
	1.2	1.09 ± 0.02	1.91	−9.10	1.07 ± 0.02	2.31	−10.54		
	6	5.71 ± 0.04	0.75	−4.85	5.71 ± 0.09	1.56	−4.83		
PC	0.2	0.18 ± 0.02	8.52	−9.00	0.21 ± 0.02	11.44	6.43	*y* = 1.77 × 10^5^*x* + 5.64 × 10^3^	0.5
	1.2	1.12 ± 0.02	1.79	−6.81	1.10 ± 0.01	1.00	−8.57		
	6	5.77 ± 0.03	0.50	−3.82	5.72 ± 0.12	0.02	−4.65		
PA	0.2	0.19 ± 0	5.11	−4.88	0.17 ± 0	1.48	−12.72	*y* = 1.98 × 10^5^*x* + 5.10 × 10^3^	0.5
	1.2	1.11 ± 0.02	1.56	−7.88	1.05 ± 0.03	3.36	−12.36		
	6	5.38 ± 0.01	0.22	−10.26	5.39 ± 0.16	3.01	−10.24		

**Table 2:** The extraction recoveries and matrix effects of GAS, HBA, PE, PB, PC and PA.

Compound	Origin (μg/mL)	Spiked (μg/mL)	Determined (μg/mL)	Recovery (%)	Matrix Effect (%)
GAS	0.7021	0.5	1.1785 ± 0.0108	95.28 ± 2.16	94.88 ± 0.02
HBA	0.1361	0.5	0.5970 ± 0.0199	92.20 ± 3.98	97.63 ± 0.62
PE	1.2966	0.5	1.8479 ± 0.0065	110.27 ± 1.29	101.93 ± 0.75
PB	2.3254	0.5	2.9022 ± 0.1082	114.98 ± 20.96	100.79 ± 0.17
PC	0.6598	0.5	1.1573 ± 0.0605	99.52 ± 12.11	106.79 ± 1.63
PA	2.6140	0.5	3.1205 ± 0.0574	101.32 ± 11.47	103.84 ± 1.07

**Table 3:** The content of GAS, HBA, PE, PB, PC, PA and the sum in extract of *G. elata*.

Content (%)	GAS	HBA	GAS + HBA	PE	PB	PC	PA	Sum of Six Compounds
Yunnan	0.356 ± 0.172	0.093 ± 0.056	0.449	0.856 ± 0.226	0.725 ± 0.204	0.196 ± 0.072	1.475 ± 0.448	3.700
Sichuan	0.378 ± 0.231	0.097 ± 0.062	0.475	0.620 ± 0.166	0.540 ± 0.158	0.128 ± 0.040	0.930 ± 0.455	2.693
Anhui	0.485 ± 0.174	0.056 ± 0.042	0.541	0.583 ± 0.076	0.546 ± 0.113	0.131 ± 0.034	1.015 ± 0.552	2.815
Zhejiang	0.410 ± 0.288	0.070 ± 0.030	0.480	0.619 ± 0.181	0.541 ± 0.150	0.128 ± 0.042	1.269 ± 0.701	3.037
Guizhou	0.353 ± 0.102	0.044 ± 0.016	0.397	0.544 ± 0.167	0.439 ± 0.164	0.127 ± 0.037	0.768 ± 0.338	2.274
Jilin	0.283 ± 0.170	0.092 ± 0.043	0.375	0.832 ± 0.143	0.561 ± 0.191	0.134 ± 0.049	0.990 ± 0.678	2.891
Shanxi	0.279 ± 0.040	0.080 ± 0.024	0.359	0.614 ± 0.076	0.569 ± 0.072	0.148 ± 0.014	1.085 ± 0.156	2.774
Xizang	0.478 ± 0.161	0.062 ± 0.030	0.540	0.799 ± 0.135	0.681 ± 0.043	0.127 ± 0.021	1.256 ± 0.252	3.402

Data, expressed as percentage of crude drug, were the mean ± SD of different batches, and each batch at three experiments.

**Table 4:** Comparison of four strategies for quality control of *G. elata.*

Strategy	Marker	Order of Quality
Pharmacopoeia	GAS and HBA or single GAS	Anhui > Xizang > Zhejiang > Sichuan > Yunnan > Guizhou > Jilin > Shanxi
Multi-markers	GAS, HBA, PE, PB, PC and PA	Yunnan > Xizang > Zhejiang > Jilin > Anhui > Shanxi > Sichuan > Guizhou
TGH-B	Total GAS, HBA	Xizang > Zhejiang > Yunnan > Anhui > Jilin > Sichuan > Shanxi > Guizhou
TH-BE	Total HBA	Xizang > Zhejiang > Yunnan > Anhui > Jilin > Sichuan > Shanxi > Guizhou

## Supplementary Materials

Supplementary materials are available online, Table S1: The content of six active ingredients in all batches of extract of *G. elata*, Table S2: Orthogonal enzymatic hydrolysis experiments, Table S3: Eight localities and quantity of *G. elata*.
