# Supplementary material for: Novel Strategies Using Total Gastrodin and Gastrodigenin, or Total Gastrodigenin for Quality Control of Gastrodia elata
Source: Molecules. 2018 Jan 29;23(2):270. doi: 10.3390/molecules23020270 (PMC6017842; doi:10.3390/molecules23020270)
Supplement: Supplementary file 1 [file molecules-23-00270-s001.pdf]

Table S1. The content of six active components, the sum of GAS and HBA and the total content of six active components in all batches of extract of *G. elata*

Content (%)	GAS	HBA	PE	PB	PC	PA	GAS and HBA	Total
Yunnan	0.620	0.062	0.823	0.822	0.265	2.102	0.682	2.727
	0.346	0.119	1.038	1.015	0.245	1.557	0.465	3.341
	0.469	0.033	0.601	0.732	0.187	1.690	0.502	1.483
	0.263	0.089	0.734	0.749	0.193	1.841	0.352	2.652
	0.186	0.198	0.933	0.604	0.112	1.140	0.384	3.924
	0.464	0.043	1.227	0.798	0.274	1.163	0.507	1.994
	0.145	0.105	0.638	0.357	0.093	0.829	0.249	2.726
	0.620	0.062	0.823	0.822	0.265	2.102	0.682	2.727
Sichuan	0.752	0.025	0.642	0.554	0.150	0.604	0.777	2.727
	0.563	0.143	0.696	0.639	0.168	1.132	0.706	3.341
	0.106	0.195	0.369	0.293	0.074	0.447	0.301	1.483
	0.238	0.099	0.862	0.468	0.106	0.880	0.337	2.652
	0.306	0.123	0.738	0.737	0.182	1.837	0.429	3.924
	0.193	0.057	0.484	0.414	0.094	0.751	0.250	1.994
	0.489	0.034	0.548	0.676	0.124	0.856	0.523	2.726
Anhui	0.708	0.026	0.566	0.689	0.151	0.940	0.735	3.081
	0.406	0.037	0.538	0.435	0.156	1.275	0.443	2.847
	0.372	0.084	0.622	0.536	0.153	1.119	0.455	2.885
	0.692	0.027	0.542	0.576	0.090	0.609	0.720	2.536
	0.270	0.124	0.744	0.687	0.178	2.006	0.394	4.009
	0.440	0.033	0.546	0.453	0.109	0.610	0.473	2.192
	0.510	0.052	0.593	0.587	0.160	1.341	0.563	3.244
	0.556	0.018	0.565	0.331	0.085	0.085	0.574	1.641
	0.677	0.027	0.463	0.608	0.092	0.653	0.705	2.521
	0.223	0.129	0.651	0.556	0.131	1.510	0.352	3.199
Zhejiang	0.918	0.024	0.302	0.285	0.057	0.182	0.942	1.768
	0.211	0.091	0.654	0.556	0.129	1.526	0.303	3.168
	0.310	0.084	0.670	0.623	0.167	1.547	0.394	3.401
	0.346	0.055	0.731	0.568	0.144	1.049	0.401	2.893
	0.265	0.096	0.739	0.672	0.141	2.039	0.361	3.953
Guizhou	0.345	0.024	0.564	0.291	0.150	0.733	0.370	2.108
	0.196	0.053	0.451	0.419	0.135	0.823	0.248	2.076
	0.480	0.035	0.310	0.274	0.062	0.268	0.515	1.429
	0.365	0.065	0.701	0.563	0.144	0.798	0.430	2.637
	0.380	0.040	0.696	0.646	0.143	1.217	0.420	3.122
Jilin	0.603	0.039	0.617	0.667	0.165	1.430	0.642	3.520
	0.214	0.106	0.768	0.349	0.106	0.311	0.320	1.853
	0.272	0.118	0.831	0.753	0.179	1.509	0.391	3.661
	0.133	0.048	0.877	0.315	0.080	0.268	0.181	1.722

	0.164	0.154	1.054	0.552	0.084	0.596	0.318	2.603
	0.310	0.087	0.843	0.732	0.188	1.825	0.397	3.986
Shanxi	0.291	0.078	0.492	0.505	0.149	1.125	0.369	2.640
	0.263	0.077	0.577	0.489	0.134	1.042	0.340	2.582
	0.307	0.127	0.676	0.629	0.159	1.204	0.433	3.101
	0.215	0.085	0.604	0.468	0.127	0.842	0.301	2.343
Xizang	0.342	0.096	0.873	0.671	0.125	1.413	0.438	3.518
	0.655	0.042	0.643	0.643	0.107	0.965	0.697	3.056
	0.437	0.048	0.880	0.728	0.149	1.390	0.485	3.632

Table S2. Orthogonal experiment of enzymatic hydrolysis.

Number	Time（h）	Rotate speed （rpm）	Enzyme concentration （U）	Peak area of HBA
1	1	400	50	468033
2	1	700	100	697095
3	1	1000	200	835066
4	2	700	50	692373
5	2	1000	100	814164
6	2	400	200	869608
7	3	1000	50	763738
8	3	400	100	831410
9	3	700	200	858158
Mean value 1	666731	723017	641381	-
Mean value 2	792048	749208	780889	-
Mean value 3	817769	804323	854277	-
extreme value	151037	81305	212895	-

Table S3. Eight localities and quantity of *G. elata*.

Locality	Quantity	Locality	Quantity
Yunnan	7	Guizhou	5
Sichuan	7	Jilin	6
Anhui	10	Shanxi	5
Zhejiang	5	Xizang	3
